# The application of stem cell sheets for neuronal regeneration after spinal cord injury: a systematic review of pre-clinical studies

**DOI:** 10.1186/s13643-023-02390-3

**Published:** 2023-11-30

**Authors:** Luchun Xu, He Zhao, Yongdong Yang, Yang Xiong, Wenqing Zhong, Guozheng Jiang, Xing Yu

**Affiliations:** Department of Orthopedics, Dongzhimen Hospital, Beijing University of Chinese Medicine, Beijing, 100700 People’s Republic of China

**Keywords:** Spinal cord injury, Stem cells, Cell sheets, Regenerative medicine, Tissue engineering, Systematic review, Animal experiments

## Abstract

**Background:**

Stem cell sheet implantation offers a promising avenue for spinal cord injury (SCI) and is currently under investigation in pre-clinical in vivo studies. Nevertheless, a systematic review of the relevant literature is yet to be performed. Thus, this systematic review aims to explore the efficacy of stem cell sheet technology in treating SCI, as indicated by experimental animal model studies.

**Methods:**

We searched PubMed, EMBASE, and Web of Science. Manuscripts that did not pertain to in vivo pre-clinical studies and those published in non-English languages were excluded. A risk assessment for bias was performed using the SYRCLE tool. Extracted data were synthesized only qualitatively because the data were not suitable for conducting the meta-analysis.

**Results:**

Among the 847 studies retrieved from electronic database searches, seven met the inclusion criteria. Six of these studies employed a complete transection model, while one utilized a compression model. Stem cell sources included bone marrow mesenchymal stem cells, stem cells from human exfoliated deciduous teeth, and adipose-derived mesenchymal stem cells. In all included studies, stem cell sheet application significantly improved motor and sensory functional scores compared to intreated SCI rats. This functional recovery correlated with histological improvements at the injury site. All studies are at low risk of bias but certain domains were not reported by some or all of the studies.

**Conclusion:**

The results of our systematic review suggest that stem cell sheets may be a feasible therapeutic approach for the treatment of SCI. Future research should be conducted on stem cell sheets in various animal models and types of SCI, and careful validation is necessary before translating stem cell sheets into clinical studies.

## Introduction

Spinal cord injury (SCI) is a condition affecting the central nervous system and carries a significant risk of disability and mortality. Furthermore, the incidence of SCI is on the rise [1–3]. Trauma is the most common cause of SCI in clinical cases [4]. SCI can be broadly categorized into primary and secondary injuries. The primary injury results from direct external forces during trauma, and subsequent secondary factors like inflammation, oxidative stress, autophagy, and cell apoptosis contribute to widespread and severe secondary cascading damage, leading to permanent loss of motor and sensory function [5–7]. Addressing spinal cord repair is challenging due to the cascade of damage that follows the primary injury, making it difficult to achieve effective treatment by targeting a single parameter [8]. Therefore, concurrently inhibiting secondary injury progression [9], promoting neuronal regeneration [10], expediting myelin sheath formation in nerves [11], and comprehensively regulating the spinal cord microenvironment [12] are pivotal yet demanding aspects of SCI treatment. Present clinical treatments such as surgery [13], glucocorticoids [14], and hyperbaric oxygen therapy [15] offer only temporary relief for secondary injury exacerbation and do not address neural regeneration. Therefore, finding an effective approach to promote neural regeneration after SCI has become a central focus in SCI research in recent years.

Stem cell transplantation has emerged as a promising method for promoting neural regeneration after SCI, with a substantial body of pre-clinical and clinical studies supporting this approach. Direct delivery of stem cells into the injury cavity formed after SCI has demonstrated significant neuroprotection and regeneration potential [16–18]. Currently, the two most commonly employed methods involve stem cell injection and transplantation onto tissue engineering scaffolds. However, the low survival rate of injected stem cells and the inflammation and immune rejection associated with biomaterial scaffold implantation have limited the application of these methods in SCI repair [19].

Cell sheet technology involves the continuous in vitro culture of high-density cells, leading to enhanced extracellular matrix secretion, resulting in a robust cell–matrix network that forms a sheet composed of cells and extracellular matrix [20]. After self-detachment, the cell sheet retains the integrity of the extracellular matrix. Compared to stem cell suspension, cell sheets significantly elevate the local seeding rate of stem cells and provide an advantageous environment for the subsequent differentiation and growth of stem cells [21]. Importantly, cell sheets enable the direct translation of stem cells into injured spinal cord tissue, eliminating the need for artificial scaffolds and effectively reducing transplantation failures caused by local inflammation and immune rejection, thereby promoting neural repair and regeneration after SCI [22].

Currently, systematic evaluations of cell sheet technology in pre-clinical studies of SCI are scarce. Therefore, this systematic review aims to explore the outcomes of stem cell sheet technology in SCI treatment based on studies conducted in experimental animal models.

## Materials and methods

The guidelines outlined in the Preferred Reporting Items for Systematic Reviews and Meta-Analyses (PRISMA) were followed throughout this investigation [23]. The research protocol was submitted to INPLASY for registration (registration number: INPLASY 202370028).

### PICO definition

In the current study, the Populations, Intervention, Comparison and Outcome (PICO) framework was defined as follows: P (Population): animals with experimentally induced SCI; I (Intervention): application of stem cell sheets; C (Comparator): stem cell suspension injection, blank, gelatine sponge or normal saline; O (Outcome): improvement in locomotor functions, sensory functions, histological neural regeneration, and occurrence of adverse effects.

### Research question

In animal models, does the application of stem cell sheets demonstrate improved outcomes for spinal cord injuries?

### Data sources

A thorough literature search of electronic databases was performed, including PubMed–MEDLINE, EMBASE, and Web of Science. The search terms used for exhaustive searches against the three databases were as follows: “cell sheet OR cell sheets OR cell aggregates OR scaffold-free” AND “spinal cord injury OR spinal cord injuries OR spinal injury OR spinal injuries OR spinal cord trauma OR spinal cord transection OR post-traumatic myelopathy OR spinal cord laceration OR spinal cord contusion.” Only studies published in English were included. We screened the reference lists of included studies for additional eligible studies not retrieved by our search.

### Inclusion and exclusion criteria

The following criteria were used to determine eligibility for inclusion in this study: (1) use of stem cell sheets; (2) in vivo studies utilizing the SCI animal model; (3) manuscripts written in English. The following types of studies were excluded: (1) manuscript designs including reviews, systematic reviews, meta-analyses, case reports, guidelines, clinical studies, and conference proceedings; (2) studies without a separate control group; (3) non-available full-text.

### Study selection

Two investigators (LX and HZ) independently scanned the titles and abstracts of all retrieved articles to determine whether the articles were pertinent to this review. Full-text articles were retrieved if either of the investigators considered the abstract potentially suitable. After retrieving the full reports of potentially relevant studies, two investigators independently assessed each study’s eligibility on the basis of the inclusion and exclusion criteria. Differences of opinion regarding study eligibility were settled by consultation with another investigator (YX).

### Data extraction

Two independent reviewers (LX and YY) extracted data from eligible studies after a thorough examination of their full texts, with any discrepancies resolved by a third investigator (XY). The data extracted from each eligible article were as follows: (1) first author; (2) publication year; (3) type of stem cell; (4) type of graft; (5) donors; (6) stem cells characterization; (7) cell sheets characterization; (8) cell differentiation at application; (9) type of animals; (10) animal model; (11) study cohorts; (12) follow-up duration; (13) outcomes. Models of SCI induction included complete transection and compression. Animal species included SD rats, Fischer 344 rats, and C57BL/6 mice. Interventions included stem cell sheets derived from human exfoliated deciduous teeth, bone marrow mesenchymal stem cell sheets, and adipose-derived mesenchymal stem cell sheets. Comparators included stem cell suspension injection, blank, gelatine sponge, and normal saline. The outcomes measured encompassed improvements in locomotor functions (assessed through BBB scores and grip strength test), sensory functions (von Frey test), histological neural regeneration (H&E staining, Nissl staining, Luxol Fast Blue (LFB) staining, IHC staining and IF staining) and the occurrence of adverse effects. In instances where relevant studies were identified, but essential information was lacking in the published article, efforts were made to contact the original authors for clarification.

### Quality assessment

Using SYRCLE’s Risk of Bias tool for animal research, two reviewers (WZ, GJ) conducted independent assessments of the quality of the articles that were included in the analysis [24]. The following ten criteria were used to assess possible bias in the enrolled studies: (1) sequence generation, (2) baseline characteristics, (3) allocation concealment, (4) random housing, (5) blinded animal intervention, (6) random outcome assessment, (7) blinded outcome assessment, (8) incomplete outcome data, (9) selective outcome reporting, and (10) other types of bias. A third reviewer was consulted to settle any disagreements of opinion that may have arisen. Each study was graded to either be of “low,” “high,” or “unclear” risk.

### Data synthesis

The data extracted from each eligible study were qualitatively synthesized within the main body of the article. Meta-analysis was not employed in this due to heterogeneity observed in the animal types, models, and interventions utilized in the primary studies. Therefore, we systematically examined and reviewed the extracted data, presenting the results in a narrative form to assess the efficacy of stem cell sheet interventions in enhancing locomotor functions, sensory functions, and histological neural aspects. We also proposed directions for future research.

## Results

### Study selection

**Fig. 1:**
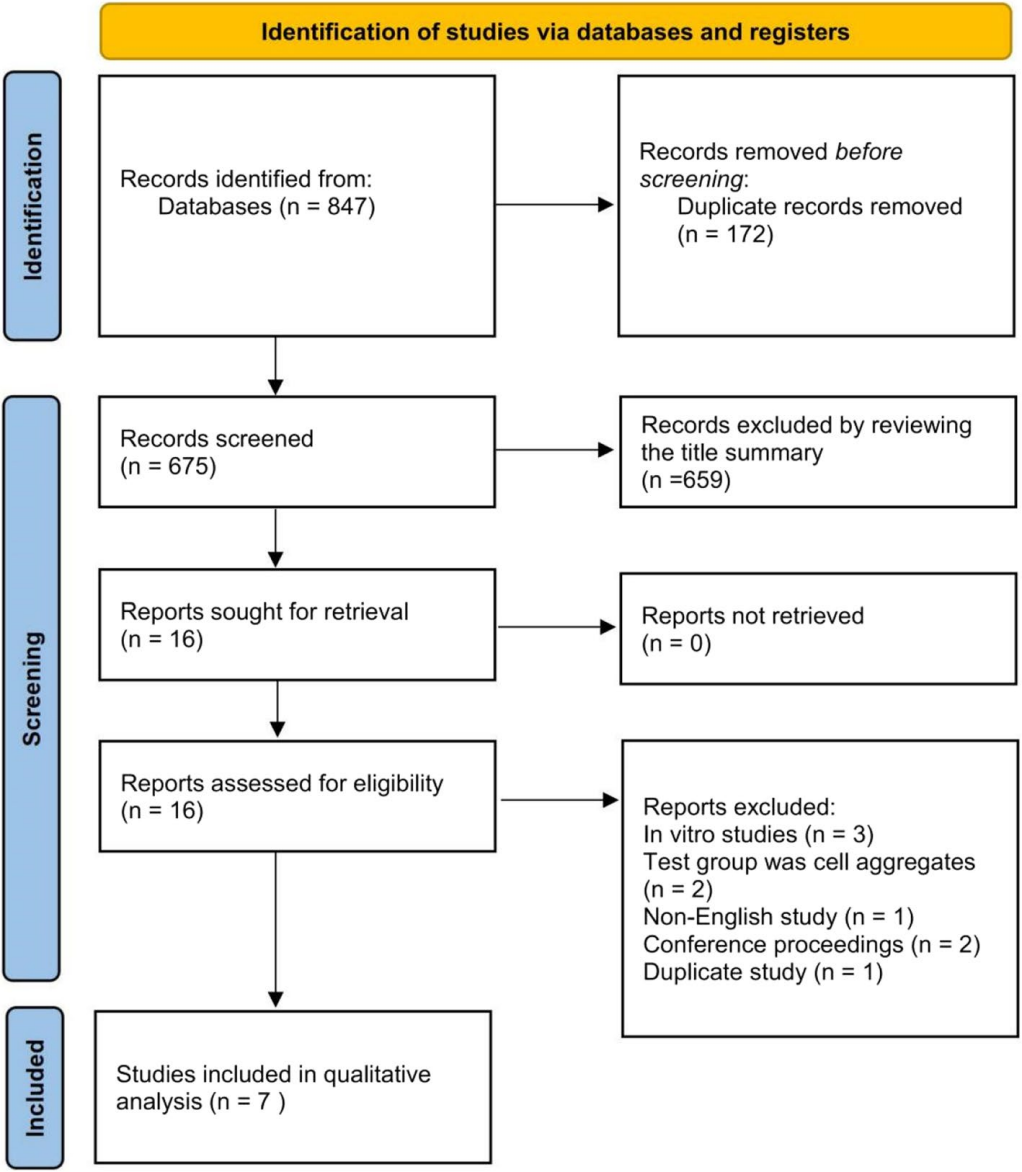
PRISMA flow diagram for identifying eligible studies

Eight hundred forty-seven records were found in the database search. After the removal of duplicates, 675 records were screened. Following the screening of titles and abstracts of identified articles, 16 articles were included as appropriate for the aim of this systematic review. After the full-text screening, 7 articles which were considered eligible according to inclusion and exclusion criteria, were finally included for qualitative analysis [22, 25–30]. Nine records might appear to meet the inclusion criteria, but after full-text screening was finally excluded due to the following reasons: in vitro studies (3 articles); test group was cell aggregates (2 articles); non-English study (1 article); conference proceedings (2 articles) and duplicate study (1 article). The PRISMA flow diagram for the search study utilized in this systematic review is presented in Fig. 1.

### Study quality assessment

**Fig. 2:**
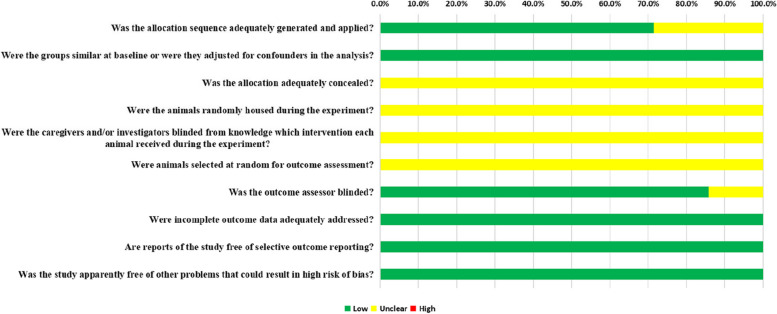
The results of the risk of bias assessment

For assessment of the risk of bias within individual studies, we used SYRCLE’s Risk of Bias tool. The outcomes of risk of bias assessments are summarized in Table 1 and Fig. 2. All studies are at low risk of bias but certain domains were not reported by some or all of the studies. According to the SYRCLE´s Risk of Bias (RoB) tool, 70 entries were obtained from the ten relevant signaling questions. Of them, 39 of the entries revealed a low RoB, 31 an unclear RoB, and no entry revealed a high RoB. Overall, five of the seven studies (71.4%) that were examined provided evidence that randomization was carried out. They only mentioned a random sequence generation but failed to report which randomization method was applied. All of the animal studies indicated that the subjects' baseline characteristics, including age, sex, and body weight, were matching. In Item 3, all the studies were considered to have an unclear RoB because they did not report any allocation concealment. All the studies were determined as unclear for random housing, investigator blinding, and random outcome assessment from knowledge of which intervention each animal received in Items 4, 5, and 6, respectively. Blinding of outcome assessors was applied in 85.7% (6/7) of studies. Regarding the attrition bias, all the studies had low RoB for incomplete outcome data in Item 8. All the studies were determined as unclear for selective reporting and other biases.

**Table 1:** Risk of bias summary: review authorssment.e studies.ondary injury processes and functiincluded study according to SYRCLEry: review authorssm

Study	SYRCLE Item									
	Item 1	Item 2	Item 3	Item 4	Item 5	Item 6	Item 7	Item 8	Item 9	Item 10
Mi et al. 2023 a [22]	Low	Low	Unclear	Unclear	Unclear	Unclear	Low	Low	Low	Low
Mi et al. 2023 b [25]	Low	Low	Unclear	Unclear	Unclear	Unclear	Low	Low	Low	Low
Chen et al. 2022 [26]	Unclear	Low	Unclear	Unclear	Unclear	Unclear	Unclear	Low	Low	Low
Li et al. 2022 [27]	Low	Low	Unclear	Unclear	Unclear	Unclear	Low	Low	Low	Low
Yamazaki et al. 2021 [28]	Low	Low	Unclear	Unclear	Unclear	Unclear	Low	Low	Low	Low
Fan et al. 2020 [29]	Low	Low	Unclear	Unclear	Unclear	Unclear	Low	Low	Low	Low
Okuda et al. 2017 [30]	Unclear	Low	Unclear	Unclear	Unclear	Unclear	Low	Low	Low	Low

SYRCLE Items: 1, sequence generation; 2, baseline characteristics; 3, allocation concealment; 4, random housing; 5, blinded animal intervention; 6, random outcome assessment; 7, blinding outcome assessors; 8, incomplete outcome data; 9, selective outcome reporting; 10, other types of bias

### Characteristics of included studies

The main characteristics of eligible studies are presented in Table 2. Five studies worked with Sprague–Dawley (SD) rats, one with Fischer 344 rats, and one with C57BL/6 mice. Complete transection (six studies) and compression (one study) were the two modes of modeling used. The spinal cord injury segments were located at T10 (five studies), T9 (one study), and T6–7 (one study). Among the included studies, the sample sizes ranged from 18 to 60. The follow-up duration in most of the studies lasted 8 weeks from the stem cell sheet implantation. Two studies ended after 60 days from the surgery, and another study after 7 weeks. The types of stem cells included stem cells from human exfoliated deciduous teeth (SHED) (two studies), adipose-derived mesenchymal stem cells (ADSCs) (one study), and bone marrow mesenchymal stem cells (BMSCs) (four studies). The transplantation types included four allogeneic and three xenogeneic studies.

**Table 2:** Summary of articles included in the systematic review

Study	Type of stem cell/type of graft/donors/stem cells characterization/cell sheets characterization	Cell differentiation at application	Type of animals	Animal model	Study cohorts	Follow-up	Outcomes
Mi et al. 2023 a [22]	SHED/Xenogenous/exfoliated deciduous teeth of clinical patients (3 donors)/flow cytometry (CD 44, CD 73, CD 90, and CD 105), Differentiation assay (Osteoblastic and adipogenic differentiation)/H&E staining, Immunofluorescence staining (collagen I, collagen III, nestin, S100, β3-tubulin, MAP2, CGRP, MBP)	Nerve induced differentiation	SD rats, 180–230 g, female, 8–10 weeks	Complete transection, T10	(1) SHED + iSHED group, *n* = 5; (2) SHED group, *n* = 5; (3) control SCI, *n* = 5; 4) sham, *n* = 5; total: 20	60 days	Locomotor function (BBB) + sensory function (Von Frey test) + axonal regeneration (IF staining) + adverse effects
Mi et al. 2023 b [25]	SHED/Xenogenous/exfoliated deciduous teeth of clinical patients (3 donors)/flow cytometry (CD 44, CD 73, CD 90, and CD 105), Differentiation assay (Osteoblastic and adipogenic differentiation) / H&E staining Immunofluorescence staining (collagen I, collagen III, nestin, S100, β3-tubulin, MAP2, CGRP, MBP)	Neurodifferentiation induced by homogenate proteins of the spinal cord	SD rats, 180–230 g, female, 8–10 weeks	Complete transection, T10	(1) hp-SHED group, *n* = 5; (2) SHED suspension group, *n* = 5; (3) control SCI, *n* = 5; 4) sham, *n* = 5; total: 20	60 days	Locomotor function (BBB + Grip strength test) + sensory function (Von Frey test) + axonal regeneration (IF staining) + adverse effects
Chen et al. 2022 [26]	ADSCs/Allogenous/female SD rats, 250–300 g/differentiation assay (osteoblastic and adipogenic differentiation)/optical microscopy, scanning electron microscopy, transmission electron microscopy, two-photon excited fluorescence microscope	Undifferentiated	SD rats, female, 250–300 g	Complete transection, T10	(1) ADSC group, *n* = 12; (2) control SCI, *n* = 12; (3) sham group, *n* = 6; total: 30	8 weeks	Axonal regeneration (H&E staining + IF staining)
Li et al. 2022 [27]	BMSCs/allogenous/male C57BL/6 mice, 6–8 weeks/flow cytometry (CD29, 45, 90), optical microscope, differentiation assay (osteoblastic and adipogenic differentiation)/H&E staining, Fluorescence microscopy (PKH67)	Undifferentiated	C57BL/6 mice, 8w	Complete transection, T10	(1) NGF-overexpressing BMSC group, *n* = 8; (2) NC-overexpressing BMSC group, *n* = 8; (3) control SCI, *n* = 8; (4) sham group, *n* = 8; total: 32	8 weeks	Locomotor function (BBB) + axonal regeneration (H&E staining + Nissl staining + IHC staining)
Yamazaki et al. 2021 [28]	BMSCs/allogenous/green fluorescent protein-expressing transgenic SD rats, 10 weeks/not reported/ELISA (HGF)	Undifferentiated	SD rats, female, 10 weeks, 250–300 g	Compression, 30 g, 1 min, T6-7	(1) BMSC group, *n* = 20; (2) BMSC cell suspension intramedullary injection group, *n* = 20; (3) control SCI, *n* = 20; total: 60	7 weeks	Locomotor function (BBB) + sensory function (Von Frey test) + axonal regeneration (H&E staining + LFB staining + IF staining)
Fan et al. 2020 [29]	BMSCs/xenogenous/Chinese big-ear white rabbits, Male and female, 180–200 g, 2 weeks/differentiation assay (osteoblastic, adipogenic, and neurogenic differentiation), Immunofluorescence staining (CD 29, CD 44, and CD 90)/scanning electron microscope, Immunofluorescence staining (collagen I, fibronectin, CD31), uniaxial tensile testing	Undifferentiated	SD rats, female, 200 g	Complete transection, T9	(1) BMSC + HUVEC group, *n* = 20; (2) BMSC group, *n* = 20; (3) control SCI, *n* = 20; total: 60	8 weeks	Locomotor function (BBB) + axonal regeneration (H&E staining + LFB staining + IF staining) + adverse effects
Okuda et al. 2017 [30]	BMSCs/allogenous/Fischer 344 rats, female, 7 weeks/not reported/cell viability assay	Undifferentiated	Fischer 344 rats, female, 8 weeks	Complete transection, T10	(1) BMSC group, *n* = 9; (2) control SCI, *n* = 9; total: 18	8w	Locomotor function (BBB) + axonal regeneration (IF staining)

*Abbreviations*: *ADSCs* Adipose-derived mesenchymal stem cells, *BBB* Basso-Beattie-Bresnahan scale, *BMSCs* Bone marrow mesenchymal stem cells, *CD* Clusters of differentiation, *CGRP* Calcitonin gene-related peptide, *ELISA* Enzyme-linked immunosorbent assay, *H&E* Hematoxylin/eosin, *HGF* Hepatocyte growth factor, *HUVEC* Human umbilical vein endothelial cell, *IF* Immunofluorescence, *IHC* Immunohistochemistry, *LFB* Luxol fast blue, *MAP2* Microtubule-associated protein 2, *MBP* Myelin basic protein, *NGF* Nerve Growth factor, *SD* Sprague–Dawley, *SCI* Spinal cord injury, *SHED* Stem cells from human exfoliated deciduous teeth, *iSHED* neural-induced SHED, *hp-SHED* SHED induced with induced with homogenate protein of spinal cord

### Locomotor function recovery

Detailed characteristics of outcomes of the included studies were summarized in Table 3. In almost all included studies the application of stem cell sheets in SCI rat models led to a significant increase in the values of BBB score (or result of grip strength test) compared with no-treated SCI rats, which indicates marked improvement of locomotor function.

**Table 3:** Summary of main outcomes and conclusions of the included studies

Study	Intervention and control groups	Locomotor function (BBB scores)	Locomotor function (Grip strength test)	sensory function (Von Frey test)	Histological analysis	Adverse effects
Mi et al. 2023 a [22]	(1) SHED + iSHED group, (2) SHED group, (3) control SCI	(1) 11.60 ± 1.14 points, (2) 7.60 ± 1.14 points, (3) 2.60 ± 0.89 points	/	(1) 100% of rats recovered sensation, (2) 80% of rats recovered sensation, (3) 20% of rats recovered sensation	Highest NF, MBP, and CGRP staining area in SHED + iSHED group; lowest cavity area and lowest number of GFAP-positive cells in SHED + iSHED group	Nonimmunotoxic to major organs such as heart, liver, spleen, lung, and kidney
Mi et al. 2023 b [25]	(1) hp-SHED group, (2) SHED suspension group, (3) control SCI	(1) 8.20 ± 0.84 points, (2) 6.40 ± 1.14 points, (3) 3.20 ± 0.84 points	(1) 235.40 ± 27.93 g, (2) 173.00 ± 16.70 g, (3) 107.80 ± 14.81 g	(1) 80% of rats recovered sensation, (2) 40% of rats recovered sensation, (3) 20% of rats recovered sensation	Highest NeuN, NF, MBP, and CGRP staining area in the hp-SHED group; lowest cavity area and lowest number of GFAP-positive cells in the hp-SHED group	Nonimmunotoxic to major organs such as heart, liver, spleen, lung, and kidney
Chen et al. 2022 [26]	(1) ADSC group, (2) control SCI	/	/	/	Highest number of β-tubulin III-positive axons and new tissue generation at the site of injury, along with the lowest cavity area, atrophy, and GFAP expression in ADSC group	/
Li et al. 2022 [27]	(1) NGF-overexpressing BMSC group, (2) NC-overexpressing BMSC group, (3) control SCI	(1) 9.40 ± 0.90 points, (2) 7.73 ± 1.28 points, (3) 3.22 ± 0.71 points	/	/	Largest number of Nissl bodies and highest positive expression of NGF, Tuj1, and GAP-43 in the NGF-overexpressing BMSC group; lowest cavity area in the NGF-overexpressing BMSC group	/
Yamazaki et al., 2021 [28]	(1) BMSC group, (2) BMSC cell suspension intramedullary injection group, (3) control SCI	(1) 11.50 ± 0.60 points, (2) 9.18 ± 0.76 points, (3) 6.99 ± 0.72 points	/	(1) 48.60 ± 12.60 g, (2) 3.19 ± 1.30 g, (3) 1.60 ± 0.59 g	Lowest lesion length, lesion volume, and GFAP-positive scar area in the BMSC group; no difference in the staining of CGRP among all groups; higher FR compared with the control group, but no significant difference compared with the BMSC cell suspension intramedullary injection group	/
Fan et al. 2020 [29]	(1) BMSC + HUVEC group, (2) BMSC group, (3) control SCI	(1) 9.58 ± 2.53 points, (2) 7.52 ± 2.29 points, (3) 7.01 ± 2.01 points	/	/	Highest number of myelinated axons and nerve fibers, the highest expression of Tuj-1, the most obvious linear staining pattern of MBP, largest LFB staining area in BMSC + HUVEC group; lowest atrophy, inflammatory response, and astrocytes, lowest expression of GFAP in BMSC + HUVEC group	Lower inflammatory reaction, no or low immunoreactivity to the host animal
Okuda et al. 2017 [30]	(1) BMSC group, (2) control SCI	(1) 5.25 ± 0.14 points, (2) 3.00 ± 0.29 points	/	/	Highest Tuj1 and GAP43 staining area, and lowest GFAP staining area in the BMSC group	/

*Abbreviations*: *ADSCs* Adipose-derived mesenchymal stem cells, *BBB* Basso-Beattie-Bresnahan scale, *BMSCs* Bone marrow mesenchymal stem cells, *CGRP* Calcitonin gene-related peptide, *FR* Fluoro-Ruby, *GAP-43* growth associated protein-43, *GFAP* Glial fibrillary acidic protein, *HUVEC* Human umbilical vein endothelial cell, *LFB* Luxol fast blue, *MBP* Myelin basic protein, *NeuN* Neuron-specific nuclear protein, *NF* Neurofilament, *NGF* Nerve growth factor, *SHED* Stem cells from human exfoliated deciduous teeth, *iSHED* Neural-induced SHED, *hp-SHED* SHED induced with induced with homogenate protein of spinal cord, *Tuj1* Neuronal class III β-tubulin

Compared to untreated and directly intramedullary injected BMSC treatment after SCI, the implantation of BMSC sheets showed significant improvement in motor function based on BBB scores (*p* < 0.01 for all comparisons). Moreover, BMSC sheets achieved more significant motor improvement within 1 week after implantation compared to the intramedullary injection group and the control group, while the intramedullary injection group did not show significant recovery until 6 weeks after transplantation (mean 11.50 ± 0.60 points for the BMSC sheets group, 9.18 ± 0.76 points for the intramedullary injection group, 6.99 ± 0.72 points for the control group) [28]. The BBB score results from another study indicated that BMSC sheets can also improve motor function after SCI, with significant differences observed compared to the control group starting from the 4th week after transplantation (mean 5.25 ± 0.14 points for the BMSC sheets group, 3.00 ± 0.29 points for the control group, *p* < 0.05 for all comparisons) [30].

Li et al. also demonstrated a significant improvement in motor function with BMSC sheets compared to the control group (mean 7.73 ± 1.28 points for the NC-overexpressing BMSC group, mean 3.22 ± 0.71 points for the control group, *p* < 0.0001). Furthermore, their study revealed that overexpressing the NGF gene in BMSC sheets using lentiviral technology led to further enhancement of motor function compared to the NC-overexpressing BMSC group (mean 9.40 ± 0.90 points for the NGF-overexpressing BMSC group, *p* < 0.0001) [27]. Moreover, culturing human umbilical vein endothelial cells (HUVECs) on the surface of BMSC sheets enabled the introduction of microvessels into the stem cell sheets, thereby providing increased blood, oxygen, and nutrient supply to the cells present in neural tissue. This approach demonstrated a significantly superior effect on the recovery of motor function compared to other comparison groups (mean 9.58 ± 2.53 points for the BMSC + HUVEC group, mean 7.52 ± 2.29 points for the BMSC group, mean 7.01 ± 2.01 points for the control group, *p* < 0.05 for all comparisons) [29].

In addition to using BMSCs for cell sheet culture, it has been shown that cell sheets formed from SHED, which exhibit stronger neurotropic properties, can also enhance motor function after SCI. Mi et al. demonstrated that by co-culturing SHED sheets with homogenate proteins of the spinal cord, SHED cells were induced to differentiate into neural cells before being transplanted into rats with spinal cord injury. On the 60th day post-SCI, a significantly greater recovery of motor function was observed compared to the control group and SHED suspension group (mean 8.20 ± 0.84 points for the hp-SHED group, mean 6.40 ± 1.14 points for the SHED suspension group, mean 3.20 ± 0.84 points for the control group, *p* < 0.001 for all comparisons). Similarly, SHED cells induced to differentiate with homogenate proteins of the spinal cord exhibited higher maximum grip strength values compared to all other study groups (mean 235.40 ± 27.93 g for the hp-SHED group, mean 173.00 ± 16.70 g for the SHED suspension group, mean 107.80 ± 14.81 g for the control group, *p* < 0.05 for all comparisons) [25]. Another study showed that co-culturing stem cell sheets formed from neuro-induced SHED with undifferentiated SHED resulted in more significant improvements in motor function scores in the grip strength test compared to single-type SHED cell sheets and the control group (mean 11.60 ± 1.14 points for the SHED + iSHED group, mean 7.60 ± 1.14 points for the SHED group, mean 2.60 ± 0.89 points for the control group, *p* < 0.001 for all comparisons) [22].

### Sensory function recovery

The recovery of motor function was consistently accompanied by sensory function recovery. Three studies evaluated sensory function recovery using the Von Frey test [22, 25, 28]. Among them, two studies utilized a complete spinal cord transection model, which often resulted in severe sensory dysfunction due to the complete disruption of spinal cord conduction pathways [22, 25]. In this model, rats typically exhibited significant sensory deficits. The third study employed a spinal cord compression model, commonly used for studying neuropathic pain after spinal cord injury [28]. In this model, impairments in ion channel function, excessive inflammatory mediators, and disruption of the descending antinociceptive serotonergic tract can lead to abnormalities in afferent nerve sensitization, causing severe neuropathic pain (hyperalgesia) in rats 1 week after modeling.

In the complete spinal cord transection model, SHED + iSHED sheets significantly increased the number and proportion of rats showing sensory function recovery compared to the control group, promoting improvements in sensory function (at a total of 20 days after surgery, 100% rats recovered sensation in the SHED + iSHED group, 80% rats recovered sensation in the SHED group, only 20% rats recovered sensation in the control group) [22], hp-SHED sheets also produced similar results, with 80% of rats recovering sensation in the hp-SHED group compared to 40% in the SHED suspension group and 20% in the control group (a total of 30 days after surgery) [25].

In the spinal cord compression model, direct intramedullary injection of BMSCs did not show improvements in neuropathic pain thresholds (hyperalgesia) within a 7-week observation period. However, significant improvement in pain hypersensitivity was observed in rats transplanted with BMSC sheets at 4 weeks post-transplantation, and this improvement became more pronounced over time (mean 48.60 ± 12.60 g for the BMSC sheets group, 3.19 ± 1.30 g for the intramedullary injection group, 1.60 ± 0.59 g for the control group, *p* < 0.01 for all comparisons) [28].

### Axonal regeneration

The functional recovery observed in the included studies was consistent with the changes observed in the histological analysis of the injury site. In most studies, the use of stem cell sheets showed significantly better results compared to the control group in terms of reduction in atrophy and presence of cavities at the injury site [26–29]. Additionally, Nissl staining results from a mouse model of complete spinal cord transection injury revealed a significant increase in Nissl bodies in mice after implantation of BMSC sheets, and a further significant increase in the number of Nissl bodies was observed after overexpression of the NGF gene in BMSC sheets.

Immunofluorescence and immunohistochemical staining demonstrated that implanted stem cell sheets promoted axonal regeneration and neuronal differentiation in the complete transection model. In most included studies, rats with implanted stem cell sheets exhibited the highest numbers of cells labeled with specific markers for neurons or axons (such as NF [22, 25], NeuN [25], Tuj-1 [27, 29, 30], GAP43 [27, 30], β-tubulin III [26], and CGRP [22, 25]) compared to the control group, indicating successful neural and axonal regeneration at the injury site. However, in the compression model, Yamazaki et al. found that the expression of FR was significantly higher compared to the negative control group but showed no significant difference compared to the BMSC cell suspension intramedullary injection group. Additionally, the study also found no difference in the staining of CGRP among all groups.

Both in the complete transection and compression model, stem cell sheets significantly promoted the regeneration of myelinated axons and inhibited the formation of glial scar. Two studies demonstrated that the area stained by luxol fast blue (LFB) was significantly larger in the groups treated with stem cell sheets compared to other comparison groups [28, 29]. Furthermore, in all studies examining the presence of myelin basic protein (MBP) using immunofluorescence, a greater number of MBP-positive myelin sheath structures were observed in animals implanted with stem cell sheets compared to other groups [22, 25], and a more pronounced linear staining pattern of MBP was also observed [29]. In studies evaluating glial scar formation using glial fibrillary acidic protein (GFAP) staining, the animals treated with stem cell sheets consistently exhibited significantly fewer GFAP-positive cells compared to other groups [22, 25, 26, 28–30]. Additionally, inducing neural differentiation of SHED sheets [22, 25] and introducing HUVECs into BSMC sheets further inhibited glial scar formation beyond the original stem cell sheets [29].

### Adverse effects

Two studies reported no immunotoxicity to major organs such as the heart, liver, spleen, lung, and kidney [22, 25]. One study observed a lower inflammatory reaction after implanting the stem cell sheet into the subcutaneous pocket, indicating either no or low immunoreactivity to the host animal [29]. However, other trials did not provide information regarding the safety of the intervention.

## Discussion

SCI is a severe traumatic disorder of the central nervous system, resulting in significant impairments in both motor and sensory functions below the level of injury [31, 32]. The condition is characterized by two distinct stages: primary and secondary injury. The primary injury occurs at the moment of trauma, causing mechanical disruption of neurons, glial cells, and nerve fibers, leading to hemorrhage and ischaemic pathological changes at the injury site [33]. The secondary cascade of injury exacerbated the initial damage, causing the cell death of various neural cells, the formation of liquefaction cavities, and the development of glial scar tissue, which acts as a barrier to tissue regeneration [34, 35]. The critical challenge in treating SCI is to inhibit secondary neuronal cell death, suppress local glial scar formation, create a conducive microenvironment for neural cell regeneration, and promote neural cell regeneration.

Stem cell transplantation has emerged as a promising approach to replenish the neuronal cells lost after SCI. Numerous studies have demonstrated that stem cell transplantation can lead to the recovery of spinal cord motor and sensory functions with relative safety [36]. However, the effectiveness of stem cell-based therapies is hampered by challenges in cell delivery and maintaining cell viability after transplantation [37]. Current methods often involve intramedullary and intravenous injection to reach the lesion site but these approaches suffer from low cell survival rates. Alternatively, the co-transplantation of stem cells loaded into artificial scaffolds has shown potential to improve therapeutic outcomes but can lead to adverse reactions, such as local inflammation and immune rejection, primarily due to the implantation of exogenous artificial materials [38, 39]. In contrast, cell sheets provide a natural structure for loading and delivering stem cells while preserving the extracellular matrix. They can be directly transplanted to the target tissue site without the need for artificial scaffolds or the transport of biomaterials. The abundant extracellular matrix in the cell sheet provides biological strength and serves as a three-dimensional network to preserve the stem cells at the site of injury, better maintaining their viability [40–42]. Additionally, some cytokines present in the extracellular matrix can play an important role in neural regeneration along with the stem cells [43].

This systematic review aimed to investigate the impact of stem cell sheet implantation on neural regeneration and functional recovery in animal models of SCI. The results from the included studies show that stem cell sheets improve the recovery of motor and sensory functions, reduce spinal cord cavitation, minimize myelin damage, and inhibit glial scar formation. Additionally, stem cell sheets enhance the number of regenerating nerve fibers at the injury site, stimulate axonal regeneration, and promote neuronal differentiation.

Furthermore, the genetic modification of stem cells and the introduction of differentiated stem cells or HUVECs into stem cell sheets have demonstrated further improvements in these aspects. For instance, overexpressing the NGF gene in BMSCs led to increased neurotrophic factor expression, alleviating histological damage and apoptosis, and improved neural regeneration [27]. Mi et al. observed that neuro-induced differentiated stem cells derived from SHED were more effective in promoting axonal myelination and suppressing glial scar formation compared to undifferentiated SHED. Co-culturing neuro-induced differentiated SHED with undifferentiated SHED provided a scaffold for cell sheet formation, resulting in improved neural nourishment and better potential for neural regeneration than single-type SHED [22]. Fan et al. addressed the challenges of ischemia and hypoxia in SCI repair by co-culturing HUVECs with BMSCs to create pre-vascularised cell sheets, facilitating the formation of microvascular networks on the cell sheets. The microvascular network on the cell sheets supported cell survival, maintained differentiation potential, and supplied the injured area with nutrients and oxygen to accelerate its repair [29]. These findings offer new strategies for stem cell-based therapy in the treatment of SCI; however, further research is warranted to validate these approaches.

The prevalent stem cell type used was BMSCs, which are widely available and commonly used in SCI treatment [44]. Moreover, the most frequently employed modeling method for SCI is the complete transection model, which involves the separation of the spinal cord and results in the loss of physiological and anatomical continuity between the rostral and caudal ends of the spinal cord. The unique characteristic of this model provides a favorable environment for exploring spinal cord regeneration, thus making it a commonly opted choice in regenerative therapies involving cell transplantation and various tissue engineering techniques [45]. Only one study explored the application of BMSC sheets in the compression model. Although BMSC sheets showed significant advantages over other control groups in terms of motor and sensory function recovery, no significant difference was observed in FR expression compared to the BMSC cell suspension intramedullary injection group. Additionally, there were no significant differences in CGRP expression compared to the other groups (BMSC cell suspension intramedullary injection group and negative control group). This could be attributed to the limited implantation space beneath the dura mater in rats owing to the incomplete transection of the spinal cord, making it difficult to slide more cell sheets into that region. Thus, larger animals should be used in future studies to overcome this limitation [28]. Spinal cord contusion is the most common type of SCI in clinical practice [46], but the efficacy of stem cell sheets in contusion models remains unexplored. Furthermore, all included studies investigated stem cell sheets in thoracic SCI models, while in clinical practice, traumatic SCI at the cervical level occurs with higher frequency [47]. To date, there are no existing studies on this topic using animal models other than rats and mice. Therefore, as research progresses, future studies should consider using larger animal models (e.g., sheep, dogs, or pigs) to provide additional support for the clinical translation of stem cell sheets.

Immune rejection is a crucial issue in tissue bioengineering. Regarding the safety of stem cell sheet technology, two studies reported nonimmunotoxicity in major organs [22, 25], and one study reported low immune reactivity to the host animal [29] without reports of severe adverse reactions. However, more research is needed in the future to further demonstrate the safety of cell sheet technology.

Despite these promising findings, our study has some limitations. First, due to the relatively new research direction of stem cell sheet technology in SCI treatment, currently available studies are limited, necessitating the inclusion of studies with larger sample sizes and higher quality in future research. Secondly, different types of stem cells, transplantation methods, animal species, and modeling approaches could introduce heterogeneity and potential bias in the study findings. Thirdly, owing to the heterogeneity of the included studies, we were unable to perform a quantitative analysis of the extracted data. Fourthly, we excluded publications written in languages other than English.

Despite these limitations, the current results support the potential of stem cell sheets in promoting motor and sensory function recovery, as well as axonal regeneration following SCI. This provides strong evidence and serves as a reference for the application of cell sheet technology in clinical settings, especially in the treatment of SCI.

## Conclusion

The results of our systematic review suggest that stem cell sheets may be a feasible therapeutic approach for the treatment of SCI. However, due to the limitations of our systematic review, which included qualitative analysis of existing data and the inclusion of studies of moderate quality, these results should be interpreted with caution. Future research should be conducted on stem cell sheets in various animal models and types of SCI, and careful validation is necessary before translating stem cell sheets into clinical studies. Furthermore, further research on stem cell sheets will allow for meta-analysis and create appropriate conditions for the clinical translation of this treatment approach.

## Data Availability

The original contributions presented in the study are included in the article, further inquiries can be directed to the corresponding author.
